# An Integrated Dialysis Pharmacometric (IDP) Model to Evaluate the Pharmacokinetics in Patients Undergoing Renal Replacement Therapy

**DOI:** 10.1007/s11095-020-02832-w

**Published:** 2020-05-14

**Authors:** Astrid Broeker, Matthias G. Vossen, Florian Thalhammer, Steven C. Wallis, Jeffrey Lipman, Jason A. Roberts, Sebastian G. Wicha

**Affiliations:** 1 Department of Clinical Pharmacy, Institute of Pharmacy, University of Hamburg, Bundesstraße 45, 20146 Hamburg, Germany; 2 Division of Infectious Diseases and Tropical Medicine, Department of Internal Medicine I, Medical University of Vienna, Vienna, Austria; 3 University of Queensland Centre of Clinical Research, Faculty of Medicine, The University of Queensland, Brisbane, Queensland Australia; 4 Department of Intensive Care Medicine, Royal Brisbane and Women’s Hospital, Brisbane, Australia; 5 University of Queensland Centre for Clinical Research, Faculty of Medicine & Centre for Translational Anti-infective Pharmacodynamics, School of Pharmacy, The University of Queensland, Brisbane, Australia; 6 Departments of Pharmacy and Intensive Care Medicine, Royal Brisbane and Women’s Hospital, Brisbane, Australia; 7 Division of Anaesthesiology Critical Care Emergency and Pain Medicine, Nîmes University Hospital, University of Montpellier, Nîmes, France

**Keywords:** adsorption, doripenem, pharmacokinetics, renal replacement therapy, teicoplanin

## Abstract

**Purpose:**

Clearance via renal replacement therapy (RRT) can significantly alter the pharmacokinetic profile of drugs. The aim of this study was (i) to improve the use of clinical trial data and (ii) to provide a model that allows quantification of all aspects of drug elimination via RRT including adsorption to dialysis membranes and/or degradation of the drug in the dialysate.

**Methods:**

An integrated dialysis pharmacometric (IDP) model was developed to simultaneously incorporate all available RRT information. The sensitivity, accuracy and precision of the IDP model was compared to conventional approaches in clinical trial simulations and applied to clinical datasets of teicoplanin and doripenem.

**Results:**

The IDP model was more accurate, precise and sensitive than conventional plasma-concentration-based approaches when estimating the clearance_RRT_ (relative bias <1%). In contrast to conventional approaches, adsorption and degradation were quantifiable using the IDP model (relative bias: −1.1% and − 1.9%, respectively). Applied to clinical data, clearance_RRT_, drug degradation (effluent-half-life_doripenem_: 13.5 h^−1^) and adsorption (polysulphone adsorption capacity_teicoplanin_: 31.2 mg) were assessed.

**Conclusion:**

The IDP model allows accurate, precise and sensitive characterization of clearance_RRT_, adsorption and degradation. Successful quantification of all aspects of clearance_RRT_ in clinical data demonstrated the benefit of the IDP model as compared to conventional approaches.

**Electronic supplementary material:**

The online version of this article (10.1007/s11095-020-02832-w) contains supplementary material, which is available to authorized users.

## Introduction

Renal replacement therapy (RRT) can alter the pharmacokinetic profile of drugs and potentially lead to therapeutic failure or increased toxicity. In order to provide reliable guidance on dose adaptation, all effects of RRT need to be quantified sensitively, accurately and precisely. The impact of RRT on the pharmacokinetic profile is usually investigated in studies containing only a small number of patients. This underlines the importance of using the clinical data from the limited patient sample undergoing RRT in the most optimal way.

Estimation of RRT clearance is often simplified in clinical practice. As a rule of thumb, drug characteristics like the protein binding, the renally-cleared fraction and lipophilicity are considered to guide an educated guess of the RRT clearance. If RRT specimens, e.g. post-filter plasma samples or dialysate samples, are available, they are not used in an integrated analysis approach, but analyzed separately. In addition, important details on RRT mode or the calculated or estimated RRT clearance are often not reported when the pharmacokinetics of patients undergoing RRT is investigated [1, 2]. Since the patients receiving RRT are often heterogenous and suffer from comorbidities, distinguishing between RRT clearance and biliary clearance or remaining renal clearance to RRT clearance can be challenging. These points emphasize the necessity to investigate dialysis processes in more detail.

### Conventional Approaches to Investigate RRT Clearance

Beyond educated guess on the clearance of drugs via RRT, we identified four conventional approaches to estimate RRT clearance: First, the plasma concentrations in patients undergoing RRT are analyzed and compared to periods, when the patients are not undergoing RRT, or to other patients not undergoing RRT [3]. Secondly, the post-filter plasma concentrations are analyzed and used to calculate dialysis clearance based on the difference between pre- and post-filter plasma concentrations and the blood flow rate [4]. Thirdly, the effluent concentrations are analyzed and used to calculate dialysis clearance based on effluent flow rates [5]. Fourthly, the cumulated effluent concentrations and the volume of the cumulated effluent, i.e. the collected effluent in the waste bag over a specified time period, are analyzed to calculate the dialysis clearance. The total amount of drug removed over time is derived and the principle of mass balance is used [6] as suggested by the FDA [7].

Even when multiple RRT specimens were measured, these data are commonly analyzed separately and compared afterwards [4, 6, 8, 9].

Using the above conventional approaches to estimate RRT clearances is associated with obvious as well as hidden restrictions. The post-filter approach considers blood flow settings only and ignores effluent settings on a mechanistical level, and vice versa for the effluent approach. This leads to difficulties when the respective other setting influences the RRT clearance. The simplification to sieving- or saturation coefficients that are linearly correlated to the resulting RRT clearance is accordingly restricted by possible non-transferability between different settings, modes or membranes. The assumption of linearity in RRT clearance to flow rates, is seldom confirmed in clinical practice [10]. The correct implementation of hematocrit, blood flow rate and filtration effect holds some challenges [11]. When the blood flow rate is used for calculation, a correction factor for hematocrit and drug concentration in the red blood cells is required as well as a correction for potential dilution and concentration effects associated with filtration processes in RRT.

### Drug Degradation in the Cumulated Effluent

Drug degradation in the cumulated effluent can potentially influence or bias the results for estimated RRT clearance when the cumulated effluent approach is used. The cumulated effluent is often measured only once per dosing interval and the stability of drugs in the effluent is typically not investigated. This might be problematic for drugs that are known to be instable, such as some beta-lactam antibiotics [12].

### Drug Adsorption to the Dialysis Membrane

Adsorption of the drug to the dialysis membrane is not considered in conventional approaches. The use of the effluent based approaches might underestimate the RRT clearance in case of adsorption leading to wrong assumptions. Highly protein bound drugs are usually considered to be not removed via RRT [8], but adsorption to the dialysis membrane might still significantly contribute to elimination of these drugs [13, 14]. Moreover, non-linear adsorption processes that are saturable or reversible, i.e. leading to time-dependent RRT clearance cannot be quantified with the conventional models. Adsorption to the dialysis membrane was observed and described over decades, recognized as potentially influencing or even requiring dose adjustments [14, 15]. But to the authors knowledge no quantitative modelling transformation from *in vitro* to *in vivo* or successful quantification using clinically available data of adsorption was performed so far.

### Purpose

Sections 1.1 to 1.3 emphasize that no one-dimensional consideration of RRT is mechanistically accurate, and RRT mode (dialysis, filtration or dia-filtration), flow rate settings, pre- or post-filter dilution, membrane material and surface area and patient characteristics can influence a drug’s RRT clearance. In this work, the focus was set on CVVHD, CVVH and CVVHDF (continuous veno-venous hemodialysis, −filtration and dia-filtration, respectively), but the principles and issues apply to all forms of RRT.

The aim of this study was (i) to improve the use of data derived in clinical trials on RRT and (ii) to propose solutions to published and hidden problems, such as drug adsorption and degradation when estimating RRT clearance.

## Materials and Methods

### Population Pharmacokinetic Modelling

For all population pharmacokinetic modelling work in this study, NONMEM® (ICON development service, Gaithersburg, MD, version 7.4) was used. The inter-individual variability of the pharmacokinetic parameters was assumed to be log-normally distributed. For the intra-individual, residual variability an additive, proportional or combined error model was used [16]. In the clinical data examples, models were evaluated by graphical and numerical criteria (goodness of fit plots, visual predictive checks and drop in objective function value (dOFV)). The LLP-SIR (log-likelihood-profiling based sampling-importance-resampling) method was employed for the assessment of parameter uncertainty, since a small number of subjects or cases was investigated [17].

### RRT Data

An integrated pharmacometric model was developed to include all RRT specimens in one model. The RRT clearance (*CL*_*RRT*_) was implemented as an additional elimination route to body clearance of the patient. The RRT clearance represented the sum of all clearance processes mediated via RRT, i.e. dialysis, filtration and adsorption to the hemofilter.

#### Post-Filter

The post-filter and dialysate specimens were included based on the mass balance equations [4]. For the post-filter specimen, the RRT clearance was parameterized as follows:

1 $$ {Q}_{blood\  adj.}={Q}_{blood}\times \left(1- Hct+ Hct\times \frac{C_{RBC}}{C_{pl(pre)}}\right) $$

2 $$ {CL}_{RRT}={Q}_{blood\  adj.}\times \frac{C_{pl(pre)}-{C}_{pl(post)}}{C_{pl(pre)}} $$

where *Hct* represents the hematocrit used to calculate the adjusted blood flow rate (*Q*_*blood adj*._) based on blood flow rate (*Q*_*blood*_), *C*_*pl*(*pre*)_ and *C*_*pl*(*post*)_ represent the plasma concentration of drug pre- and post-filter, respectively. A correction factor for blood cell concentration (*C*_*RBC*_) was required depending on the drug specific blood to plasma ratio [4]. For filtration processes, a correction term for post-filter measurements is required when a fluid removal rate is used and in CVVH or CVVHDF when sampling is done before adding the post-filter replacement fluid as described previously [9].

3 $$ {C}_{pl(post)\kern0.2em corr.}={C}_{pl(post)\kern0.1em meas.}\times \frac{Q_{blood\kern0.1em adj.}-\left({Q}_{RF\kern0.2em post}+{Q}_{FRR}\right)}{Q_{blood\kern0.2em adj.}} $$

Here, *Q*_*RF post*_ represents the flow rate of the replacement fluid added post-filter and *Q*_*FRR*_ the fluid removal rate. A more robust way to correct for post-filter measurements is the normalization to the hematocrit, when pre- and post-filter hematocrit measurements are available.

#### Effluent

For the effluent specimen, the RRT clearance was calculated as follows:

4 $$ {CL}_{RRT}={Q}_{effl.}\times \frac{C_{effl.}}{C_{pl(pre)}} $$

5 $$ {Q}_{effl.}={Q}_{dial}+{Q}_{RF\  pre}+{Q}_{RF\  post}+{Q}_{FRR} $$

where *Q*_*effl*._ represents the total effluent flow rate, *Q*_*dial*_, *Q*_*RF pre*_ and *Q*_*RF post*_ represent the dialysate, the pre- and post-filter replacement fluid flow rate, respectively, *c*_*effl*._ represents the concentration of drug in the effluent and *C*_*pl*(*pre*)_ represents the pre-filter plasma concentration.

#### Cumulated Effluent

For the cumulated effluent specimen, RRT clearance was calculated using the amount of drug in the cumulated effluent

6 $$ {amt}_{cum.\kern0.5em effl.}={\int}_{t_1}^{t_2}{CL}_{RRT}\times {c}_{pl(pre)}\  dt $$

The amount in the effluent compartment (*amt*_*cum*. *effl*._) was modelled as an output compartment in a similar fashion as suggested for urine measurements [16].

### Simulation Study

For the stochastic simulation and estimation (SSE) study, the automated SSE tool by PsN [18] was used with *n* = 1000 simulations. Clinical trials with different extents of RRT clearance, ranging from 0.001% to 100% of the total body clearance, were simulated. The developed IDP model (Eqs. 7–10) was employed for the clinical trial simulations including all concentration-time data (pre- and post-filter plasma, effluent and cumulated effluent). The model parameters were (re-)estimated from the concentration-time data of the simulated trials using the reduced model (only pre-filter plasma data), the post-filter model, the effluent model, the cumulated effluent model (pre-filter plasma data and one of the RRT specimens, respectively) or the IDP model (using all data simultaneously). This simulation and (re-)estimation allowed the assessment of model performance in simulated (virtual) clinical trials in order to draw conclusions on the model’s performance on real clinical data. Furthermore, the simulated truth is known, e.g. the true dialysis clearance and further pharmacokinetic parameters, and allows the calculation of performance metrices. All five approaches were compared regarding their accuracy (relative bias, rBias), precision (relative root mean squared error, rRMSE) and power to detect RRT clearance.

#### Study Design

A dataset including 10 dialysis and 10 non-dialysis patients was used for the SSEs. Four pharmacokinetically different antibiotics (piperacillin, tigecycline, colistin and linezolid) served as example drugs and typical dosing schemes were simulated. In all simulation examples the blood cell concentration was assumed to be zero.

A typically used rich sampling schedule for the first and for the fifth dose in pre- and post-filter plasma and effluent (t = 0.5 h, 1 h, 2 h, 4 h, 6 h, 8 h; t = 1 h, 2 h, 4 h, 6 h, 8 h, 12 h for 8 h and 12 h dose interval, respectively) was chosen [5]. Cumulated effluent concentration and volume were collected over the dosing interval and determined once at the end of a dosing interval. For the non-dialysis patients, RRT clearance was set to zero and only pre-filter plasma concentrations with the same sampling schedule were included.

Residual variability was set conservatively to avoid overestimation of power or precision to 25% for the concentration measurements (pre- and post-filter plasma, (cum.) effluent) and to 10% for the volume measurements. Further details on the drug examples used for the simulations are provided in the supplementary material (Table S1).

CVVHD was chosen for the SSEs with settings of *Q*_*blood*_= 10 L/h (167 mL/min), Hct = 0.3 and *Q*_*Dial*_= 3.0–3.3 L/h, reflecting typical settings in CVVHD [1].

#### Drug Degradation in the Cumulated Effluent

Degradation of drug in the cumulated effluent was simulated using the piperacillin example. A degradation rate constant of 0.03 h^−1^ corresponding to a half-life of approximately 24 h was simulated to mimic instable beta-lactams [19]. Accuracy and precision of the estimated parameters using the conventional cumulated effluent approach as compared to using the IDP model was investigated. Only with the IDP model it was feasible to account for drug degradation in the cumulated effluent, being informed by time-dependent differences in the estimated clearance between effluent and cumulated effluent. The dosing interval and the sampling interval of effluent were set to 8 or 12 h. The RRT clearance was simulated to be as high as the body clearance (3 L/h).

#### Drug Adsorption to the Dialysis Membrane

The example drug colistin was used to investigate accuracy and precision of the conventional effluent and cumulated effluent approaches as compared to the IDP model, when adsorption to the dialysis membrane was simulated. The conventional approaches were not accounting for adsorption, while the IDP model was estimating the adsorption processes. The RRT clearance was assumed to be as high as the body clearance in the simulations (2 L/h). When non-reversible adsorption was simulated, the fraction of the RRT clearance mediated by adsorption was set to 65% of the RRT clearance without saturation. When reversible adsorption was simulated, the fraction of the RRT clearance mediated by adsorption was set to 95% of the RRT clearance and the reversible adsorption rate constant to 0.07 h^−1^ resulting in lower, non-linear adsorption clearance. The simulated fraction of RRT clearance mediated via adsorption was considered plausible with respect to the fraction of RRT clearance mediated via adsorption of teicoplanin (see results section).

### Case Studies with Pre-Clinical and Clinical Datasets

#### Doripenem

A previously published pharmacokinetic study of 12 patients undergoing CVVHDF receiving doripenem was investigated as clinical data example for the application of the IDP model [6]. 195 pre-filter plasma concentration, 194 post-filter plasma concentration, 71 effluent concentration, 40 cumulated effluent concentration and 31 volume of the effluent measurements were included in the analysis. No samples below the quantification limit were reported. The blood flow rate was 200 mL/min, the dialysate flow rate 1000 mL/h and the replacement fluid rate 1000 or 2000 mL/h with variable fluid removal rate. A polyacrylonitrile filter (AN69 Nephral ST 200, Gambro Lundia AB, Lund, Sweden) was used. Further details of the study can be found in the publication by Roberts *et al*. [6]. The general model structure was resumed as described by Roberts *et al*. [6], where pre-filter plasma concentrations and amounts in cumulated effluent had been used. For the dialysis data, the IDP model was used to describe all data simultaneously. For the post-filter plasma samples, a correction factor was used, since the post-filter plasma samples were obtained pre-addition of replacement fluid (Eq. 3). Possible adsorption to the dialysis membrane with and without a capacity limitation term was investigated, being parameterized by a potentially time-dependent clearance estimated from the pre-filter-post-fitler clearance and pre-filter-effluent clearance. The hematocrit was set to 0.30 for all patients [20].

Moreover, the IDP model was used to quantify potential degradation of doripenem in the cumulated effluent, being parameterized by the difference in the estimated clearance using the pre-filter-effluent clearance and the cumulated-effluent-based clearance.

#### Teicoplanin

An *in vitro* study investigating RRT of teicoplanin in bovine blood was evaluated using the IDP model. 51 pre-filter plasma samples and 45 post-filter plasma samples were included. 45 effluent samples were collected which were all below the lower limit of quantification (5 mg/L), which was considered in the analyses by comparison to simulations after estimation. A blood concentration of 25 mg/L using a volume of 2.2–2.5 L was studied. The blood flow rate was set to 250 mL/min and the dialysate flow rate to 500 mL/min without filtration. In five experiments, a polysulphone membrane (*n* = 3: FX80, Fresenius Medical Company, Austria, *n* = 2: F60 S, Fresenius Medical Company, Austria), and in one experiment a triacetate membrane (Ni 21 e, Surflux-21E, Nipro Corporation, Japan) was used. The IDP model was used to describe the RRT clearance and adsorption to the dialysis membranes was evaluated.

An *in vivo* case study with two patients receiving teicoplanin undergoing CVVHF [14] was evaluated using the IDP model. One patient received 1000 mg every 24 h and samples were taken in the first dosing interval. The other patient received 400 mg teicoplanin every 24 h and samples were taken in the second dosing interval. The replacement fluid rate was 75 and 60 mL/h; blood flow rate was 200 and 150 mL/h, respectively. A polyamide membrane (Gambro FH66D, Gambro, Austria) was used. 11 pre-filter plasma samples, 11 post-filter plasma samples and 2 effluent samples were included in the analyses. No samples below the lower limit of quantification were reported. The IDP model was used to describe the RRT clearance. It was tested for adsorption to the dialysis membranes.

## Results

### The Integrated Dialysis Pharmacometric (IDP) Model

#### Model Structure

**Fig. 1:**
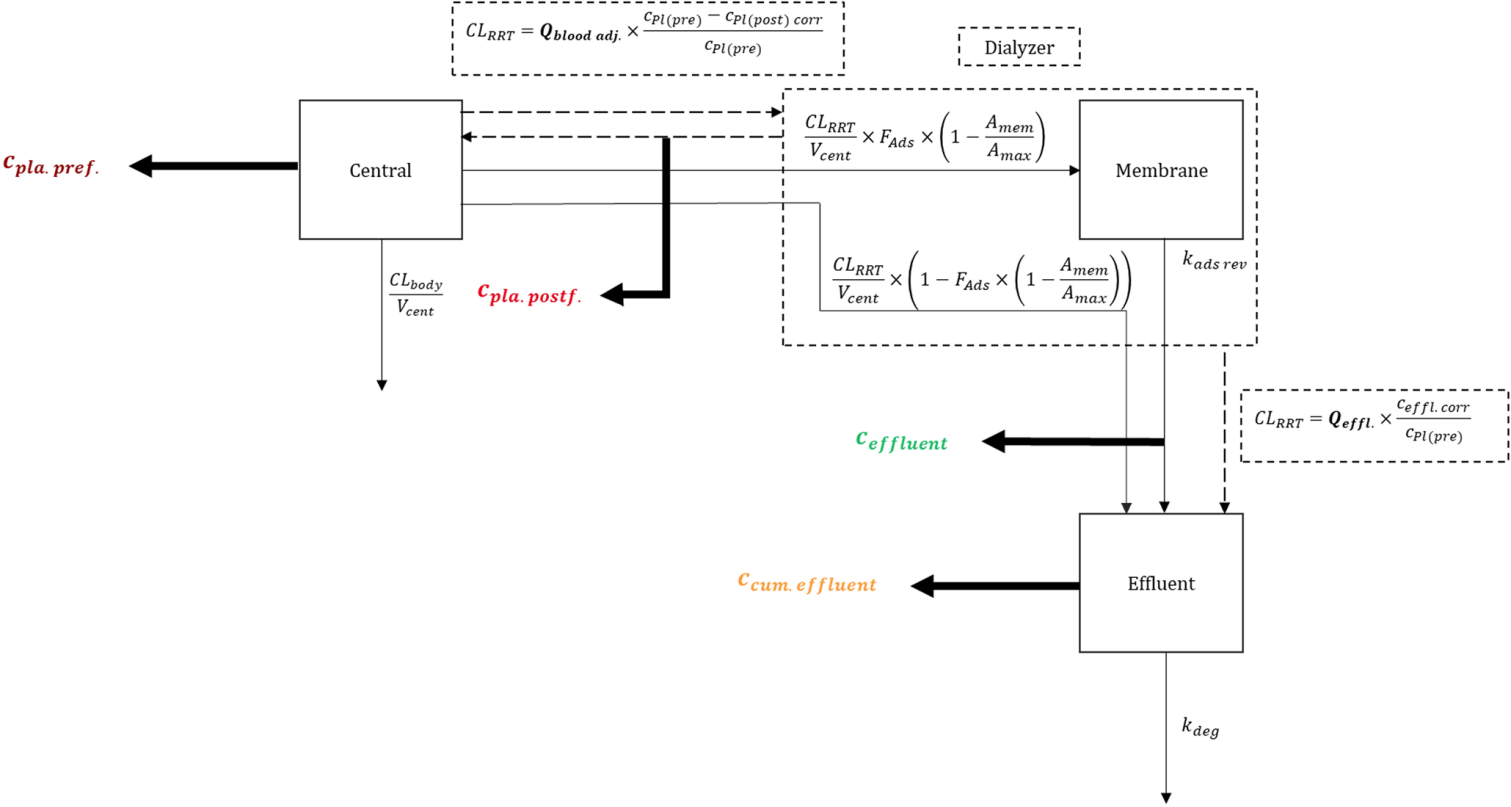
Schematic overview of the IDP model with reversible adsorption. Bold arrows indicate measurements, thin arrows mass transfer, dashed arrows mechanistic flows of the dialyzer. ***c***_***pl***(***pre***)_, ***c***_***pl***(***post***)_, ***c***_***effl.***_: concentration of pre- and post-filter plasma, of effluent and cumulated effluent; ***CL***_***body***_: Clearance mediated by the human body; ***CL***_***RRT***_: total RRT clearance; ***k***_***deg***_: degradation rate; corr: corrected; cum.: cumulated ***F***_***Ads***_, ***Ads***_***max***_ and ***k***_***ads rev***_: fraction, maximal and reversible rate of adsorption; ***Q***_***effl.***_, ***Q***_***bood adj.***_: effluent and adjusted blood flow rate.

The IDP model allowed to include measured pre- and post-filter plasma concentrations, effluent and cumulated effluent concentrations and volume of the cumulated effluent simultaneously within a single model (Fig. 1). These measurements are included as dependent variables, while flow rates, hematocrit, and blood to plasma ratios are usually independent variables, but the integrated structure allows the estimation of particular variables, if required, e.g. the dialysis flow rate based on the volume measurements. The integration of all measurements allowed to quantify adsorption to the dialysis membrane and degradation in the cumulated effluent. The total clearance mediated via RRT, i.e. dialysis, filtration and adsorption processes, is given as *CL*_*RRT*_. An example for a NONMEM control stream and a NONMEM dataset specification is provided in the supplementary material (Appendix model 1, simulation_pip.csv). Implemented into an ordinary one compartment pharmacokinetic model, the IDP model is given as follows:

7 $$ \frac{d{A}_1}{dt}=-\frac{CL_{RRT}}{V_{cent}}\times {A}_1-\frac{CL_{body}}{V_{cent}}\times {A}_1 $$

8 $$ \frac{d{A}_2}{dt}=\frac{CL_{RRT}}{V_{cent}}\times {A}_1\times {F}_{Ads}\times \left(\ 1-\frac{A_2}{Ads_{max}}\right)-{k}_{ads\  rev}\times {A}_2 $$

9 $$ \frac{d{A}_3}{dt}=\frac{CL_{RRT}}{V_{cent}}\times {A}_1\times \left(1-{F}_{Ads}\times \left(\ 1-\frac{A_2}{Ads_{max}}\right)\right)+{k}_{ads\  rev}\times {A}_2-{k}_{deg}\times {A}_3 $$

10 $$ \frac{d{A}_4}{dt}={Q}_{effl.} $$

The drug amounts in the central compartment (*A*_1_), adsorption compartment representing the binding capacity of the dialysis membrane (*A*_2_) and effluent compartment (*A*_3_) were included in the IDP model as described in Equation 7, Equation 8 and Equation 9, respectively, where *CL*_*body*_ represents the clearance mediated by the body; *A*_1 − 3_ the drug amounts in the compartments 1–3; *F*_*Ads*_, *Ads*_*max*_ and *k*_*ads rev*_ the fraction of RRT clearance mediated via adsorption, maximal amount adsorbed and reversible rate of adsorption, respectively; *k*_*deg*_ the degradation rate in the cumulated effluent. The volume of the cumulated effluent (*A*_4_) was described using *Q*_*effl*._ as shown in Eq. 10. The adsorption compartment (*A*_2_) was empty and opened with the first dose of drug and reset with every recorded filter change in a patient. The effluent compartment (*A*_3_) and the volume of the effluent compartment (*A*_4_) were set to zero at the start of effluent collection and reset with every change of effluent bag.

#### Drug Adsorption to the Dialysis Membrane

Possible adsorption to the dialysis membrane was included in the model by adding an adsorption compartment representing the binding capacity of the dialysis membrane (*A*_2_). It described the adsorbed amount of the drug (*A*_2_) as a fraction (*F*_*ads*_) of the amount of drug cleared by dialysis. This expression can be expanded by a term describing a maximal amount adsorbed to the membrane (*A*_*adsmax*_) and/or by a term that describes the reversibility of the adsorption by the rate *k*_*ads rev*_. Only the amount that is removed by dialysis and not adsorbed to the membrane is collected in the effluent compartment. The IDP model enables flexible estimation of the adsorption process allowing inclusion of covariates, e.g. membrane material on the maximal amount adsorbed to the membrane, and adaption in the number of estimated parameters based on quantifiability and plausibility.

Notably, the RRT clearance is split in a fraction mediated by dialysis and/or filtration, and a fraction mediated by adsorption. The notation shown in Eqs. 7–10 represents a stable total RRT clearance due to an increase of dialysis and filtration efficacy, when the adsorption maximum was reached. Another parameterization represents a stable fraction of RRT clearance mediated by dialysis and filtration not depending on the amount of drug bound to the membrane. In such a scenario, the total RRT clearance affecting the central compartment was accordingly not stable over time, but dependent on the amount of drug bound to the membrane (*A*_2_):

11 $$ \frac{d{A}_1}{\mathrm{d}t}=-\frac{CL_{RRT}}{V_{cent}}\times {A}_1\times \left(\left(1-{F}_{Ads}\right)+\left({F}_{Ads}\times \left(\ 1-\frac{A_2}{Ads_{max}}\right)\right)\right)-\frac{CL_{body}}{V_{cent}}\times {A}_1 $$

The adaptions to the other equations for this scenario can be found in the Appendix model 1.

#### Drug Degradation in the Cumulated Effluent

In order to estimate possible degradation of the drug, a degradation rate constant *k*_*deg*_ was included in the cumulated effluent compartment (Eq. 9).

#### Observations

The predictions of the concentration measurements for the RRT specimens pre- and post-filter plasma, effluent and cumulated effluent and for the volume of the cumulated effluent were obtained as shown in Eq. 12–16. Notably, no assumption on the volume of post-filter plasma and effluent was required to incorporate these measurements in the IDP model and accordingly no assumptions on the volume and no ‘virtual’ compartments were required.

12 $$ {c}_{plasma\  pre- filter}=\frac{A_1}{V_{cent}} $$

13 $$ {c}_{plasma\  pos\mathrm{t}- filter}=\frac{A_1}{V_{cent}}-\frac{A_1}{V_{cent}}\times \frac{C{L}_{RRT}}{Q_{blood\kern0.2em adj.}}\times \frac{Q_{blood\kern0.2em adj.}}{Q_{blood\kern0.2em adj.}-\left({Q}_{RF\kern0.2em pos t}+{Q}_{FRR}\right)} $$

14 $$ {c}_{effl uent}=\frac{A_1}{V_{cent}}\times \frac{CL_{RRT}}{Q_{effl.}}-\frac{d{A}_2}{dt}\times \frac{1}{Q_{effl.}} $$

15 $$ {c}_{cumulated\kern0.5em effluent}=\frac{A_3}{V_{effl.}} $$

16 $$ {c}_{volume\ cumulated\ effluent}={A}_4 $$

The post-filter plasma concentration was calculated based on the mass balance equations. For post-filter plasma sampled before addition of the replacement fluid, a correction as described in Eq. 3 was employed. However, especially in CVVH and CVVHDF dilution effects are complex. In order to ensure no omitted dilution effects, the determination of the hematocrit in pre- and post-filter samples might be useful.

The measured effluent concentration was influenced by the drug adsorbed to the dialysis membrane (Fig. 1). Therefore, the change of drug amount per time in the membrane compartment ($\frac{d{A}_2}{dt}$, Eq. 8) per dialysate flow was included in Eq. 14.

In contrast to calculating amounts from measured cumulated effluent concentration and volume before performing the analysis, cumulated effluent concentration and volume are integrated in the analysis. The amount in the effluent (Eq.9) was linked to volume and concentration of the cumulated effluent, which are directly measurable in a dialysis study.

The volume of the cumulated effluent was expressed as dialysate flow rate over time (Eq.10) and linked to the observations as described in Eq. 16. The drug concentration in the cumulated effluent was described based on the prediction for the volume and amount (Eq. 15).

### The Simulation and Estimation Study

#### Power to Detect RRT Clearance

**Fig. 2:**
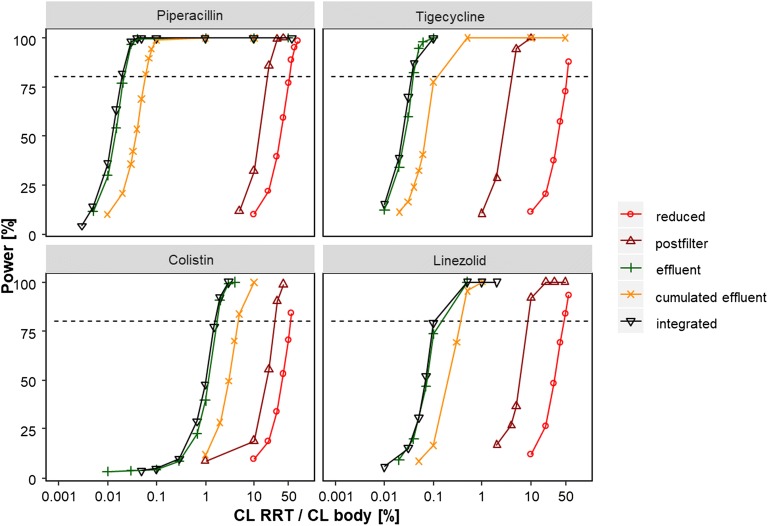
Power to detect RRT clearance by reduced, post-filter, effluent, cumulated effluent and IDP approach in four drug examples. Horizontal line indicates 80% power.

The power to detect RRT clearance with the reduced, the post-filter, the effluent, the cumulated effluent and the IDP model is presented in Fig. 2. The reduced model, using only pre-filter plasma data of dialysis and non-dialysis patients, had the lowest power to detect RRT clearance. For instance for piperacillin, an RRT clearance as high as 60% of the body clearance (1.8 L/h *vs*. 3 L/h) would be detectable with a power >80%. For the post-filter model, using pre- and post-filter plasma concentrations, an RRT clearance of >20% of the body clearance was needed to be detected with a power >80% for piperacillin. The effluent based approaches, using the effluent and the cumulated effluent, respectively, were much more sensitive and detected an RRT clearance >0.03% and 0.06% of the body clearance with a power >80% in the piperacillin example, respectively. The IDP model, using all RRT specimens at a time, was the most sensitive approach, where already an RRT clearance of only 0.02% of the body clearance resulted in >80% power for piperacillin. The same pattern was observed in the other drug examples (tigecycline, colistin and linezolid).

#### Accuracy and Precision

**Fig. 3:**
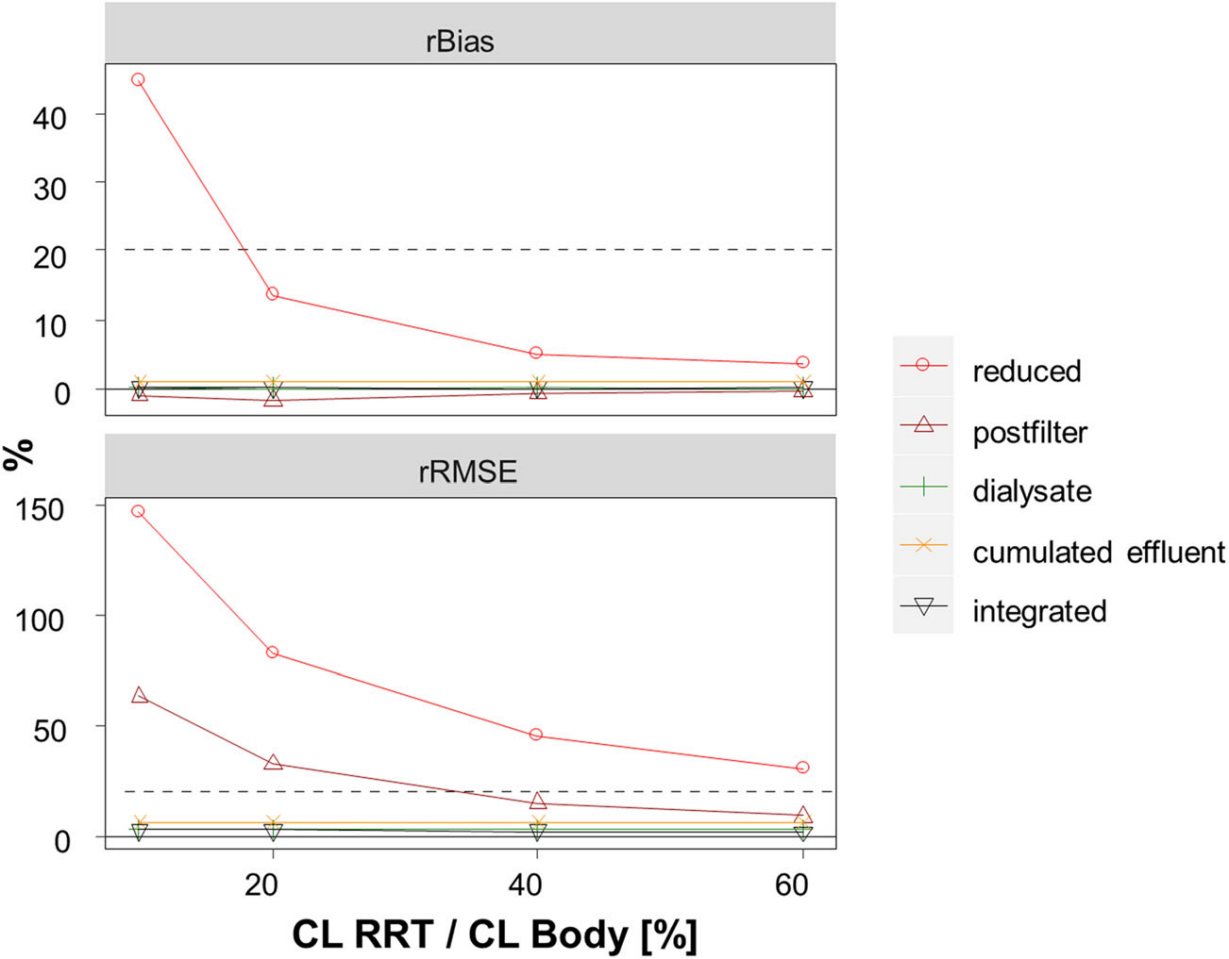
Accuracy and precision of estimated RRT clearance by reduced, post-filter, effluent, cumulated effluent and IDP approach. Horizontal line indicates 20% relative bias (rBias) and 20% relative root mean squared error (rRMSE).

The accuracy and precision of RRT clearance assuming no degradation or adsorption determined via rBias and rRMSE using all five approaches is presented in Fig. 3. The RRT clearance was estimated least precisely by the reduced model and by the post-filter model (rBias: 13.4% and − 1.6%, rRMSE: 82.3% and 32.7% rRMSE for an RRT clearance 20% of the body clearance with the reduced and post-filter model, respectively). Generally, with increasing RRT clearance, the estimation of the RRT clearance was more precise by the plasma-based approaches. The (cumulated) effluent based models gave accurate and precise estimates of the RRT clearance over all investigated drug examples (rBias <1%, rRMSE<7%). The IDP model was slightly more precise as compared to the other approaches and accurate over all tested scenarios.

#### Drug Degradation in the Cumulated Effluent

Drug degradation in the cumulated effluent was simulated and its influence on accuracy and precision of the estimated RRT clearance was evaluated for the cumulative effluent approach and for the IDP model. A simulated degradation half-life of 24 h in a dosing interval of 8 and 12 h lead to a rBias of −12.1% and − 19.7% and an rRMSE of 13.4% and 20.3%, respectively, when RRT clearance was estimated with the cumulated effluent approach. The IDP model resulted in accurate and precise estimates of the RRT clearance (rBias: −0.07% and − 0.07%, rRMSE: 3.9% and 3.9%, for an 8 and 12 h dosing interval). The degradation rate constant *k*_*deg*_ was quantifiable with an rBias of −1.9% and − 1.1% and an rRMSE of 42.0% and 26.0% for an 8 and 12 h dosing interval, respectively.

#### Drug Adsorption to the Dialysis Membrane

Adsorption to the dialysis membrane was simulated and its influence on accuracy and precision of the estimated RRT clearance was evaluated for the effluent and cumulated effluent approaches as well as for the IDP model. The irreversible adsorption led to imprecise and biased estimates of the RRT clearance using the effluent approaches (rBias: −65.0% and −64.8%, rRMSE: 65.0% and 64.8% for the effluent and cumulated effluent approach, respectively). The reversible adsorption caused imprecise and biased estimates of the RRT clearance for the effluent approach (rBias −42.2%, rRMSE 42.3) as well as for the cumulated effluent approach (rBias −35.9%, rRMSE 36.3%). For irreversible adsorption, the structural model parameter estimates (i.e. *V*_*cent*_, *CL*_*body*_) were unbiased except for body clearance with both effluent approaches. For reversible adsorption, the effluent model resulted in biased estimates for *V*_*cent*_ (rBias: 15.2%) as well and for *CL*_*body*_ (rBias: 16.3%), but the structural model was not affected using the cumulated effluent approach except for body clearance (rBias 22.3%). The IDP model gave accurate and precise estimates of the RRT clearance for the irreversible and reversible case (0.49% and 0.71% rBias, 18.7% and 6.05% rRMSE, respectively). No bias occurred in the structural model (rBias<5%) and the adsorption process was quantifiable with the IDP model. The reversible and irreversible fraction of adsorption were estimated with an rBias of 0.07% and − 1.9% and an rRMSE of 0.65% and 12.5%, respectively.

### Pre-Clinical and Clinical Dataset Examples

#### Doripenem

**Fig. 4:**
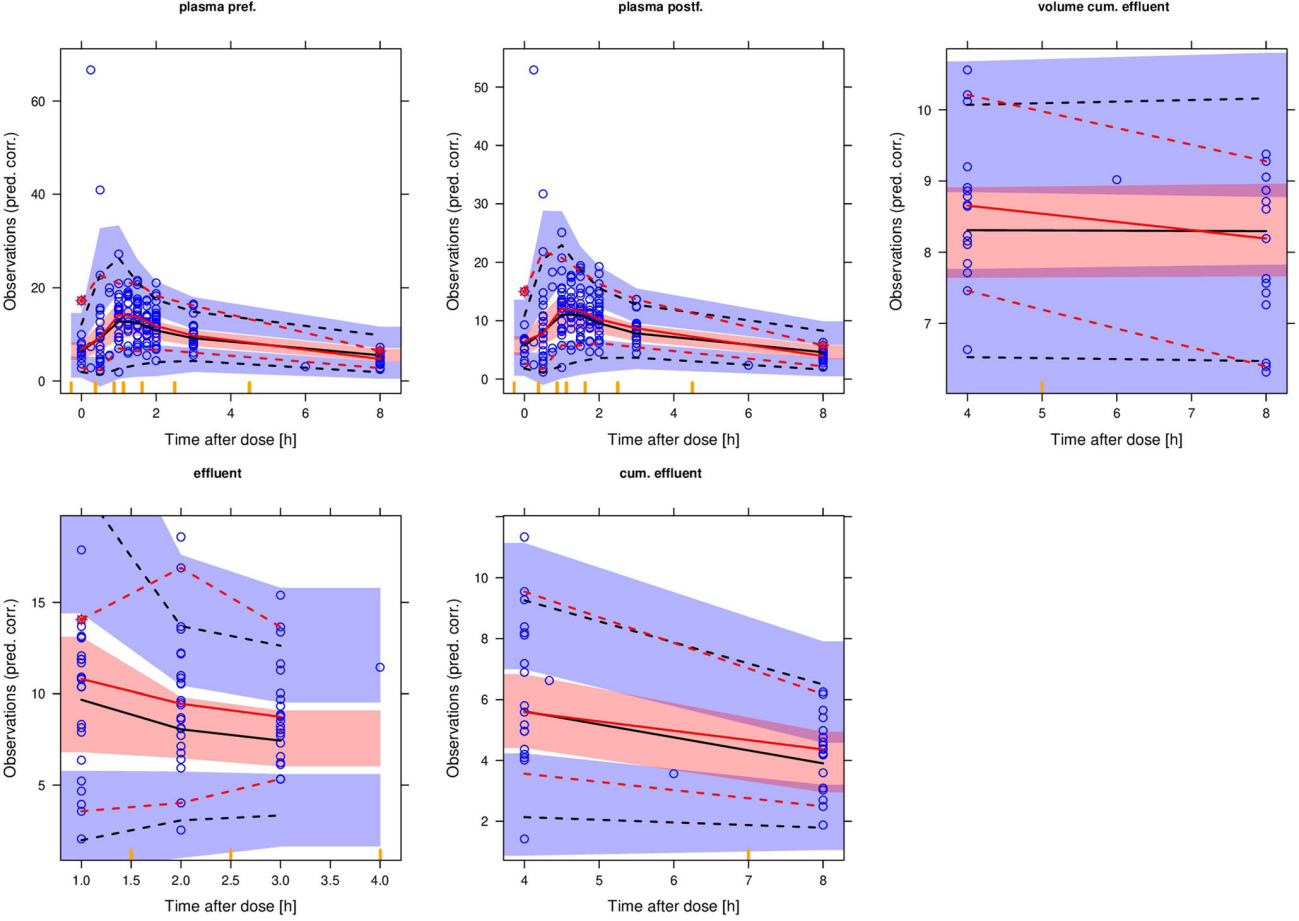
Visual-predictive checks on doripenem concentrations in pre- and post-filter plasma, effluent and cumulated effluent and on volume of the cumulated effluent in the 5th, 50th and 95th percentile with the shaded area describing the 90% confidence interval.

The IDP model was successfully applied to the doripenem clinical dataset (Supplementary Table S2). Pre- and post-filter plasma, effluent, cumulated effluent and volume of the effluent data were all described simultaneously within one model as visualized in the visual predictive checks (Fig. 4). The RRT clearance was estimated to 2.46 L/h and the total effluent flow rate, here the sum of dialysis rate, replacement rate and fluid removal rate, was used as a proportional covariate relationship on effluent flow rate normalized to 3 L/h. A degradation half-life of 13.5 h for doripenem in the cumulated effluent was estimated and improved the model fit significantly (dOFV: −9.16, *p* = 0.0025). A capacity limited binding of 164 mg and a fraction adsorbed of 24.1% were estimated and resulted in a dOFV of −14.65. The total RRT clearance including adsorption mediated clearance did not decrease when the capacity limit was reached.

When comparing the results obtained from the IDP model to the conventional models, similar estimates for RRT clearance were provided by the post filter model, where adsorption processes are included but not differentiated from the RRT clearance mediated via dialysis and filtration processes (2.46 L/h). The effluent approach, where only RRT clearance mediated via dialysis is captured, provided lower results (2.07 L/h). The cumulated effluent model provided an even lower RRT clearance of 1.91 L/h, deviating by 8.4% from the effluent approach and by 28.8% from the IDP approach, since degradation of doripenem is omitted leading to under-estimation of the RRT clearance.

The inclusion of the effluent flow rate as covariate on RRT clearance resulted in a dOFV of −25.09 (*p* < 0.1^−5^) for the IDP model and of −15.5, −17.7 and − 18.4 in the conventional approaches, post-filter, effluent and cumulated effluent, respectively, and reduced IIV CL_RRT_ in all approaches (e.g. from 25.4% to 6.7% for the IDP model).

#### Teicoplanin

**Fig. 5:**
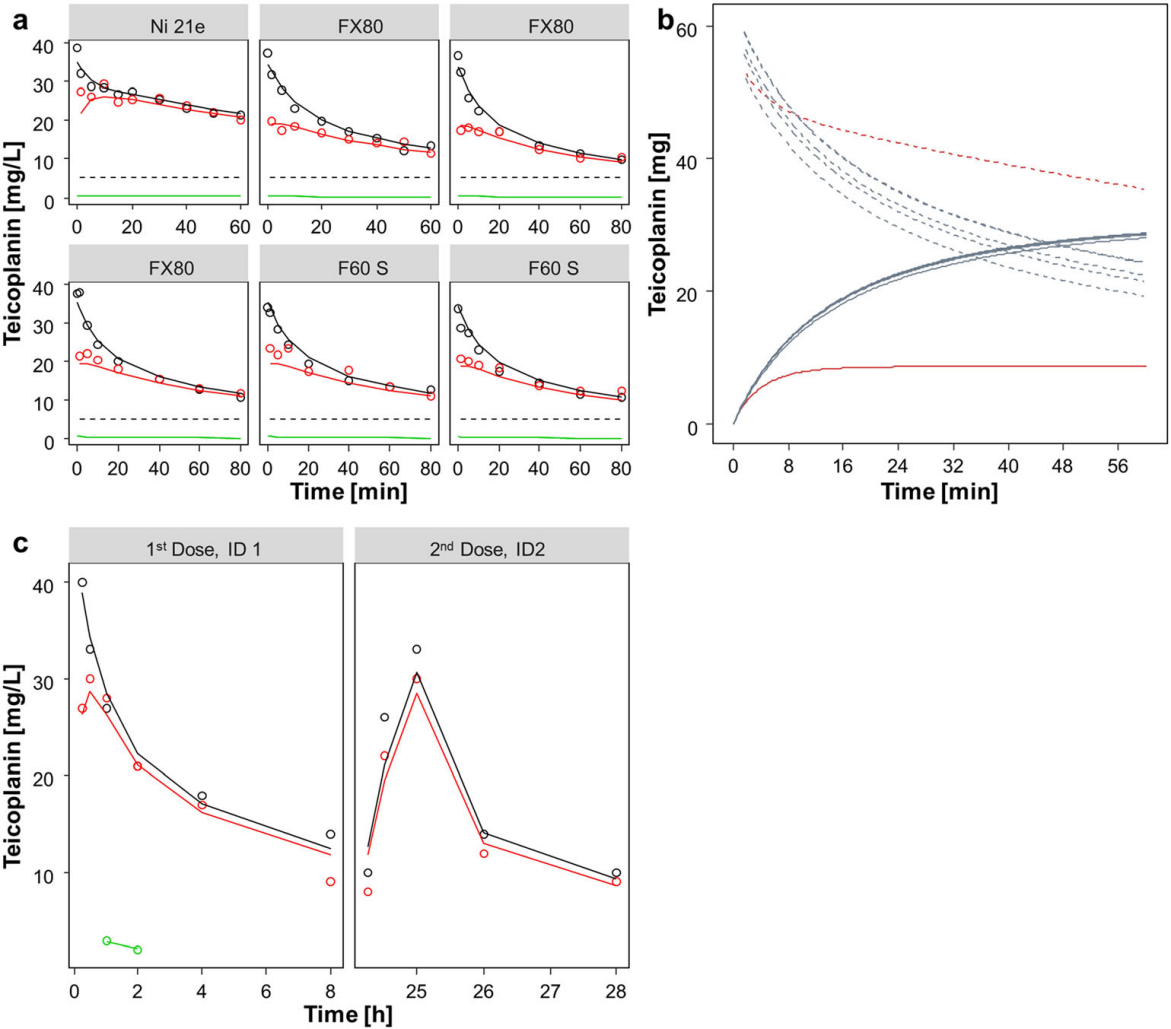
**(a**) individual fits teicoplanin *in vitro*, points: observation, solid lines: individual model fit, black: pre- filter plasma, red: post-filter plasma, green: effluent, dashed line: lower limit of quantification. (**b**) mass balance teicoplanin in vitro, solid lines: amount adsorbed to the dialysis membrane, dashed line: amount of drug in plasma, red: triacetate membrane, blue: polysulphone membrane. (**c**) individual fits teicoplanin *in vivo*, points: observation, solid lines: individual model fit, black: pre- filter plasma, red: post-filter plasma, green: effluent.

The IDP model was successfully applied to the teicoplanin *in vitro* data (Supplementary Table S3) and the graphical evaluation of the individual fits showed good alignment of the model prediction with the observed data (Fig. 5a). An RRT clearance of 4.59 L/h was estimated, which was changing over time. The RRT clearance was mainly mediated via adsorption and only a minor part of the drug was removed constantly via the dialysis processes. This was reflected in the mass balance analyses (Fig. 5b). The adsorption was characterized with a fraction of the RRT clearance mediated via adsorption of 0.891 and a capacity limitation term. The drug removed via dialysis processes was stable over time and not dependent on the drug adsorbed to the membrane. The maximal adsorption capacity was dependent on the material of the membrane and was 8.6 mg (5.1 mg - 11.8 mg, 95% confidence interval) and 31.2 mg (26.5 mg - 36.7 mg) for triacetate and polysulphone membranes, respectively.

When the IDP model was applied to the data of the teicoplanin *in vivo* case study patients, a similar pattern as in the *in vitro* study was observed (Supplementary Table S2, Fig. 5c). The RRT clearance was in a similar range (4.91 L/h *in vivo* as compared to 4.59 L/h *in vitro*) and also mainly mediated via adsorption (fraction of the RRT clearance mediated via adsorption of 0.896 and 0.891 *in vivo* and *in vitro*, respectively). The drug removed via dialysis and filtration processes was not dependent on the drug adsorbed to the membrane, but stable over time. Using the capacity limitation term, a maximal capacity of 40.3 mg was estimated for the polyamide membrane.

## Discussion

### The Integrated Dialysis Pharmacometric (IDP) Model

The IDP model unites all RRT samples, i.e. pre- and post-filter plasma, effluent and cumulate effluent concentrations and volume measurements of the effluent within a unique model. All settings regarding RRT type (continuous/intermitted, dialysis, filtration, dia-filtration), drug properties (protein binding, red blood cell concentration), corrections for pre- and post-filter replacement fluid or fluid removal rates and membrane types are considered with the IDP model.

For the IDP model, more data as compared to the conventional approaches are needed. As shown in the clinical examples, also reduced datasets can be evaluated with the IDP model, but dependent on which RRT specimen is missing at the cost of accuracy and precision or not describing adsorption or degradation (Table 1). The costs of collecting additional data to the pre-filter plasma samples can be deemed acceptable, given that these trials are usually performed in small patient collectives and increase of the patient sample size is not an option. Hence, as much information as possible should be derived from these trials and even samples from compartments that are commonly considered waste (effluent, cumulated effluent) can be highly informative.

**Table 1:** Study Design Recommendations for Application of the IDP Model Approach and Contribution of Specimen to Derive Quantitative Information. Hct: Hematocrit

Quantitative information gained from IDP approach	Requirements	Specimen measured						
		Pre-filter Plasma	Post-filter Plasma	Effluent	Cumulated effluent	Volume cumulated effluent	Hct pre-filter (once)	Hct postfilter (frequently)
Accurate and precise determination of RRT clearance Quantification of adsorption to the dialysis membrane Quantification of drug degradation in the cumulated effluent Verify and correct for dilution processes occurring in CVVH(D)F Estimation of effluent flow rate		X	X	X	X	X	X	X
No correction for dilution processes possible	No filtration processes (only CVVHD)	X	X	X	X	X	X	
No estimation of effluent flow rate possible		X	X	X			X	X
No correction for dilution processes possible No estimation of effluent flow rate possible	No filtration processes (only CVVHD)	X	X	X			X	
No quantification or detection of adsorption processes possible	Adsorption excluded	X		X	X	X		
No quantification or detection of adsorption processes possible No quantification of degradation in the cumulated effluent possible	Adsorption excluded, Degradation determined	X			X	X		

The IDP model can be beneficial to guide therapy by providing a better understanding and quantification of RRT processes potentially allowing improved or precision dosing. Its benefit can potentially be expanded in clinical applications, e.g. in TDM processes. Modern software supporting TDM could potentially allow parallel evaluation of plasma and effluent measures alongside information on the RRT like membrane type or flow rates.

Some inconsistency of the usage of the mass balance derived conventional approaches to calculate RRT clearance were discussed by Atkinson [11], who emphasized using the adjusted blood flow rate corrected for hematocrit and red blood cell concentration in the post-filter approach. Notably, no adjustment to the effluent was required in case of pre-filter dilution or post-dilution. A correction term for pre-filter dilution as described by Pea *et al*. [2], characterized the translation of efficacy of a post-dilution system to a pre-dilution system. Pre-filter dilution is less efficient due to a dilution of the blood before it enters the dialysis membrane. Notably, when concentrations were determined in a pre-dilution system, this correction factor was not needed and would even violate the mass balance equation. The measured concentrations in the effluent in a pre-filter dilution system will be lower as compared to the concentration in a post-filter dilution system and accordingly the differences are addressed in the mass balance equation without correction factor.

Some steps towards simultaneous analyses were taken by Leuppi-Taegtmeyer *et al*. [21], but were not fully in line with the underlying mechanisms of RRT. The pharmacokinetic model used by Leuppi-Taegtmeyer *et al*. was incorporating post-filter plasma measurements and effluent measurements within one model and described RRT clearance based on the mass balance. However, the model required assumptions on the fictive volume of filter and cartridge, while the IDP model incorporates all measurements based on the actual flow rates independent of the hemofilter “distribution” volume and is therefore more in line with the true mechanisms of RRT. The characterization and quantification of adsorption processes was not discussed by Leuppi-Taegtmeyer, even though colistin has been described to display relevant adsorption to the hemofilter [22, 23]. The IDP model allowed to describe and quantify irreversible, reversible and capacity limited adsorption.

### The Simulation and Estimation Study

#### Power, Accuracy and Precision

The IDP model was superior regarding precision, accuracy and power especially to the reduced model, where only pre-filter plasma concentrations were included. The reduced model provided a biased estimate for the RRT clearance. This can be explained by the distribution of the estimates for RRT clearance (Supplementary Fig. S4), where the lower boundary was 0 but no upper limit was given, which resulted in a skewed distribution of estimates (mean: 0.43 L/h, median: 0.36 L/h, true: 0.3 L/h). The post-filter model was less sensitive and precise as compared to the approaches using (cumulated) effluent data. When the differences between pre- and post-filter plasma are small, these might be hidden due to the residual error of both specimens. For the (cumulated) effluent approaches, RRT clearance is detectable more reliably since drug concentrations exceeding the range of the additive error strongly support the identification of an RRT clearance. This indicates that detectability of drug and hence the lower limit of quantification of the bioanalytical method influences the power to detect RRT processes. Adsorption or degradation processes potentially reduce the power to detect dialysis clearance since the less sensitive plasma-based approaches are of higher relevance in such scenarios. Overall, regarding accuracy, precision and power, an approach using (cumulated) effluent data as in the IDP model is beneficial.

#### Drug Degradation in the Cumulated Effluent and Drug Adsorption to the Dialysis Membrane

When degradation or adsorption was included, the IDP model was superior regarding accuracy and precision of the estimated RRT clearance as compared to the effluent based approaches. For the cumulated effluent model, the impact of degradation on the RRT clearance depended on the collection interval and the stability of drug in the effluent. However, using the IDP model, RRT clearance was estimated accurately and precisely over all scenarios and the degradation half-life was directly estimable within the model.

For the (cumulated) effluent approaches, adsorption lead to biased RRT clearance estimates. Using the conventional effluent approach, even the estimated structural model parameters (i.e. *V*_*cent*_, *CL*_*body*_) were affected as well in case of reversible binding processes to the dialysis membrane. The underlying assumption in the effluent approach, that pre-filter plasma concentrations and effluent concentrations are in a constant ratio not changing over time (Equation 4) lead to over-estimation of volume of distribution and under-estimation of total clearance (Supplementary Fig. S5). Instead, the IDP model accounted for the changing ratio of pre-filter plasma to measured effluent concentration (Eq. 14) and provided unbiased estimation of the structural model parameters, the RRT clearance and the adsorption processes in all tested scenarios.

### Pre-Clinical and Clinical Dataset Examples

#### Doripenem

The IDP model described pre- and post-filter plasma, effluent and cumulated effluent concentrations and the volume of the cumulated effluent simultaneously and successfully characterized degradation in the cumulated effluent. A capacity limited adsorption to the dialysis membrane was estimated. With respect to the surface area, similar adsorbed amounts of the same material, polyacrylonitrile filters, was found for ticarcillin *in vitro* (up to 85 mg, 0.6 m^2^ surface area) [24], while 167 mg adsorbed doripenem was estimated for 1.05 m^2^ surface area with the IDP model in the present study. The RRT clearance determined with the IDP model was more in line with the RRT clearance determined with the post-filter approach while effluent based approaches would lead to smaller RRT clearances not capturing adsorption processes. The estimated degradation rate constant for doripenem (half-life of 13.5 h) was in line with degradation observed in destabilizing conditions in the literature such as elevated temperature, oxidative stress or exposure to UV radiation (half-life of 12 h and 27 h *vs*. >100 h in destabilizing *vs*. stabilizing conditions, respectively) [12, 25, 26]. Hence, the estimated degradation was in the range of previously determined half-lives and suggested destabilizing conditions in the effluent. The dOFV and the reduction of IIV in RRT clearance due to covariate inclusion of effluent flow rate as a covariate in all approaches underlined a dependency of the actual RRT clearance to the dialysis settings following the mass balance equations. However, it remains unclear if RRT clearance is in all cases linearly changing with effluent and/or blood flow rates. Saturable dialysis and filtration processes are possible scenarios as well as non-linearity through adsorption processes or protein binding [2]. Employing the IDP model in a systematic investigation across different dialysis settings could elucidate such correlations. For doripenem, this was not observed and the linearity between RRT clearance and effluent flow rate was considered plausible.

The integrated analysis of concentration in the cumulated effluent and volume of the cumulated effluent instead of previously calculating the amount of drug in the cumulated effluent allowed the use of all concentration measurements (*n* = 40), even when no volume measurement (*n* = 31) was recorded.

#### Teicoplanin

The *in vitro* study of teicoplanin revealed a capacity limited binding of teicoplanin to the membrane. The maximal amount adsorbed to the membrane was depending on the membrane type, being in line with findings of Shiraishi *et al*. [13]. However, only the IDP model allowed for quantification of the fraction and maximal amount of drug adsorbed to the membrane while adsorption or capacity limitation could be explored only graphically with the conventional approaches. Hence, wrong conclusion would be drawn from the calculations based on the conventional approaches not accounting for adsorption. The fraction of teicoplanin removed via dialysis was very small, which was in line with the previous studies [27].

The adsorption and filtration behavior of teicoplanin was in line with the *in vivo* results, suggesting that the behavior of a drug *in vitro* described with the IDP model is a sufficient base to transfer adsorption and dialysis behavior of a drug to clinic.

Protein binding plays an important role when the RRT clearance is only estimated based on drug characteristics, since it is assumed that only unbound drug can be dialyzed or filtrated. The integrated model allows direct assessment of the RRT clearance without assumptions on the efficacy in dialysis and filtration processes based on the unbound fraction. Moreover, elimination processes, that are independent of dialysis and filtration, e.g. adsorption to the dialysis membrane, can be quantified. RRT clearance of highly protein bound drugs, e.g. teicoplanin presenting a protein binding of >90% in patients with normal serum albumin concentrations [28], can be quantified overcoming the assumption of low RRT clearance due to the high protein binding acknowledging adsorption.

### Study Design Recommendations

For future studies, we recommend to consider which RRT specimen might provide useful input to the analysis. An overview of implications, requirements and the respective RRT specimens to obtain is provided in Table 1.

The post-filter RRT specimen can provide valuable information and is itself not influenced by drug degradation. Moreover, drug adsorption to the dialysis membrane is intrinsically included or detectable when the post-filter approach is used to calculate an RRT clearance. However, the post-filter approach was less sensitive and less precise as compared to the effluent approaches and is highly dependent on precise knowledge of the blood flow rate and the hematocrit, where undetected variabilities or missing information would lead to erroneously calculated RRT clearances. In contrast to the effluent flow rate, which can be verified by the cumulated volume in the effluent bag, no direct verification is possible for blood flow. In addition, for methods using filtration or replacement fluids (CVVH and CVVHDF), a determination of the hematocrit in each sample, i.e. in the pre- and post-filter sample, is recommended to verify the occurring dilution processes.

The effluent specimen allowed accurate, precise and sensitive estimation of the RRT clearance when no adsorption of the drug to the dialysis membrane occurred. However, the effluent-bases approach is highly sensitive to the effluent flow rate and therefore undocumented or unmeasured discrepancies would lead to erroneous results in the estimated RRT clearance.

The cumulated effluent approach was providing accurate, precise and sensitive estimation of RRT clearance at a low sample number. However, adsorption to the dialysis membrane and undetected drug degradation in the cumulated effluent can lead to biased results. Also, time-and concentration-dependencies in the RRT clearance cannot be detected. The cumulated effluent approach is independent to flow rates when the volume is determined and the volume information can even support flow rate estimation for the effluent approach.

When degradation of the drug in the cumulated effluent was not excluded in previous *in vitro* studies or quantified in stability tests, the additional collection of effluent samples and the application of the IDP model is recommended.

When adsorption to the dialysis membrane was not excluded for the respective membrane type, the collection and the use of all RRT specimens is highly recommended. The post-filter approach including hematocrit measurements is required to quantify the extent of total RRT clearance, while the effluent specimen allows time dependent insight on the binding process and the cumulated effluent provides robust, flow independent mass balance information.

When degradation and adsorption are excluded in previous or *in vitro* studies, dialysis based approaches are recommended and the sampling size can be reduced by using the cumulated effluent approach.

### Limitations

This study was focusing on continuous RRT and no proof of concept for intermitted RRT in patients was provided. This was considered reasonable, since patients undergoing continuous RRT are often more vulnerable, the dosing after RRT is not possible like for intermitted patients and in-patient comparisons of pharmacokinetic with and without RRT is often hard to get. However, the underlying theory of RRT is similar and thus, our findings might be transferable.

Since the main focus was on RRT clearance, body clearance was estimated as one parameter and it was not distinguished between non-renal and renal clearance. However, the IDP model can easily be extended to estimate non-renal and renal clearance separately as demonstrated by Roberts *et al*. [6].

Due to a lack of hematocrit measurements in the doripenem dataset, a hematocrit of 0.3 in all patients was assumed, which matches the hematocrit in patients undergoing RRT. Yet, as the hematocrit influences the calculated post-filter clearance, use of a different hematocrit will lead to a different adsorption profile and hence the estimated amount of doripenem bound the dialysis membrane needs to be interpreted with caution. Moreover, undetected dilution effects cannot be excluded. We therefore want to underline the importance of measuring the hematocrit both in pre- and post-filter samples to parameterize the pre-post-filter clearance correctly and directly account for dilution effects in the measured post-filter samples.

For the teicoplanin *in vivo* example only two patients with full pharmacokinetic data were available and it can therefore only be seen as an indicator for transferability of the IDP approach from *in vitro* to *in vivo* and not as a base for reliable clinical conclusions.

## Conclusion

To conclude, the IDP model allowed the accurate, precise and sensitive determination of RRT clearance and was superior to conventional approaches. Adsorption and degradation processes can be quantified with the IDP model, which was not possible with the conventional approaches. The IDP model was successfully applied to *in vitro* and *in vivo* clinical data and degradation of the drug in the cumulated effluent and adsorption to the dialysis membrane was quantifiable. Accordingly, the IDP model is a promising approach to better make use of clinical trial data and will provide quantitative insights in removal of drug via RRT.

## Electronic supplementary material

ESM 1 (TXT 5.62 kb)

ESM 2 (CSV 7.83 kb)

ESM 3 (DOCX 76.7 kb)

## Abbreviations

CVVHD: Continuous veno-venous hemodialysis
CVVH: Continuous veno-venous hemofiltration
CVVHDF: continuous veno-venous hemodiafiltration
dOFV: Drop in objective function value
Eq.: Equation
IDP model: Integrated dialysis pharmacometric model
LLP-SIR: log-likelihood-profiling based sampling-importance-resampling
rBias: relative Bias
rRMSE: Relative root mean squared error
RRT: Renal replacement therapy
SSE: Stochastic simulation and estimation
