# Supplementary material for: An Integrated Dialysis Pharmacometric (IDP) Model to Evaluate the Pharmacokinetics in Patients Undergoing Renal Replacement Therapy
Source: Pharm Res. 2020 May 14;37(6):96. doi: 10.1007/s11095-020-02832-w (PMC7225193; doi:10.1007/s11095-020-02832-w)
Supplement: Supplementary file 3 — (DOCX 76.7 kb) [file 11095_2020_2832_MOESM3_ESM.docx]

**Supplementary Table S1** Dosing regimen and pharmacokinetic parameters used for the stochastic simulation and estimation study

| **Drug** | **Piperacillin** | **Tigecycline** | **Colistin** | **Linezolid** |
| --- | --- | --- | --- | --- |
| Dose [mg] | 3000 | 50 (loading dose 100) | 33 | 600 |
| Infusion duration [h] | 0.5 | 1 | 0.5 | 0.5 |
| Dose interval | 8 h / 12 h | 12 h | 8 h | 12 h |
| Volume of distribution [L] | 30 | 300 | 30 | 40 |
| Body clearance [L/h] | 3 | 15 | 2 | 7 |
| IIV body clearance [% CV] | 50 | 50 | 50 | 50 |
| IIV volume of distribution [% CV] | 50 | 50 | 50 | 50 |
| Add. error (cumulated) effluent [mg/L]^a^ | 0.1 | 0.002 | 0.075 | 0.1 |

^a^corresponds to 20% of typical assay quantification limits.

**Supplementary Table S2** Final parameter estimates of the doripenem model. Parameter uncertainty was determined via sampling importance resampling using log-likelihood profiling as proposal distribution (LLP-SIR)

| Description | Doripenem (adsorption) | |
| --- | --- | --- |
| **Parameter** | **Estimate** | **LLP-SIR** |
| CL_Body_ [L/h] | 3.1 | 2.39 - 3.88 |
| V1 [L] | 14.4 | 9.19 - 21.92 |
| CL_RRT_ [L/h]^*^ | 2.46 | 2.23 - 2.68 |
| Inter-compartmental clearance [L/h] | 20.9 | 16.58 - 25.7 |
| V2 [L] | 21.7 | 16 - 27.8 |
| k_Absortpion_ | 2.66 | 1.36 - 4.51 |
| Correction Q_Effl_ | 0.902 | 0.87 - 0.93 |
| k_degradation_ | 0.0515 | 0.02 - 0.08 |
| Fraction_RRT mediated via adsorption_ | 0.241 | 0.16 - 0.32 |
| Adsorption capacity hemofilter [mg] | 164 | 37.5 - 408.4 |
| Residual variability prefilter [%] | 12.5 | 9.85 - 15.31 |
| Residual variability prefilter [mg] | 1.11 | 0.82 - 1.43 |
| Residual variability postfilter [%] | 13.7 | 11.05 - 16.39 |
| Residual variability postfilter [mg] | 0.912 | 0.65 - 1.17 |
| Residual variability effluent [%] | 17.6 | 12.98 - 22.97 |
| Residual variability effluent [mg] | 0.625 | 0.12 - 1.25 |
| Residual variability cum. effluent [%] | 9.89 | 4.57 - 15.57 |
| Residual variability cum. effluent [mg] | 0.531 | 0.26 - 0.83 |
| Residual variability volume cum. eff. [mg] | 1.15 | 0.91 - 1.47 |
| IIV CL_Body_ [% CV] | 42.43 | 30.22 - 69.21 |
| IIV V1 [% CV] | 73.89 | 50.47 - 113.31 |
| IIV CL_RRT_ [% CV] | 6.93 | 3.23 - 11.81 |
| IIV V2 [% CV] | 39.5 | 19.75 - 69.32 |
| IIV K_a_ [% CV] | 97.93 | 20.62 - 185.64 |
| IOV K_a_ [% CV] | 131.91 | 76.47 - 208.08 |

^*^The covariate relationship was implemented as described in Eq. 1.

| ${CL}_{RRT}=\theta_{1}\times\frac{Q_{dial}+Q_{RF post}+Q_{FRR}}{3000}$ | (  1) |
| --- | --- |

**Supplementary Table S3** Final parameter estimates of the teicoplanin in vitro an in vivo study. Parameter uncertainty was determined via sampling importance resampling using log-likelihood profiling as proposal distribution (LLP-SIR)

| Description | Teicoplanin in vitro | | Teicoplanin in vivo | |
| --- | --- | --- | --- | --- |
| **Parameter** | **Estimate** | **LLP-SIR** | **Estimate** | **LLP-SIR** |
| CL_RRT_ [mL/min] | 76.5 | (67.1 - 84.5) | 69.8 | (46.6 – 94.6) |
| Fraction_Adsorbed_ | 0.891 | (0.85 - 0.93) | 0.896 | (0.83-0.93) |
| Adsorption capacity triacetate [mg] | 8.6 | (5.1 - 11.8) |  |  |
| Ads. capacity polysulfone FX80/F60S [mg] | 31.2 | (26.5 - 36.7) |  |  |
| Ads. capacity Triacetate membrane Ni 21e [mg] |  |  | 40.3 | (2.21 - 154) |
| Correction V1 | 1.06 | (1.03 - 1.11) |  |  |
| CL_Body_ [L/h] |  |  | 2.51 | (2.03 - 3.02) |
| V1 [L] |  |  | 13.2 | (5.72 - 36.8) |
| Inter-compartmental clearance [L/h] |  |  | 8.22 | (4.9 - 12.4) |
| V2 [L] |  |  | 14.8 | (10.99 - 18.54) |
| Residual variability prefilter [%] | 6.2 | (4.97 - 7.93) | 11.6 | (8 - 23) |
| Residual variability postfilter [%] | 10.4 | (7.96 - 14.0) | 13.8 | (9 - 22) |
| Residual variability effluent [%] |  |  | 8.2 | (4 - 22) |
| Inter-individual variability correc. V1 [%] | 2.3 | (0.53 - 5.01) |  |  |
| Inter-individual variability V1 [% CV] |  |  | 66 | (29 - 125) |


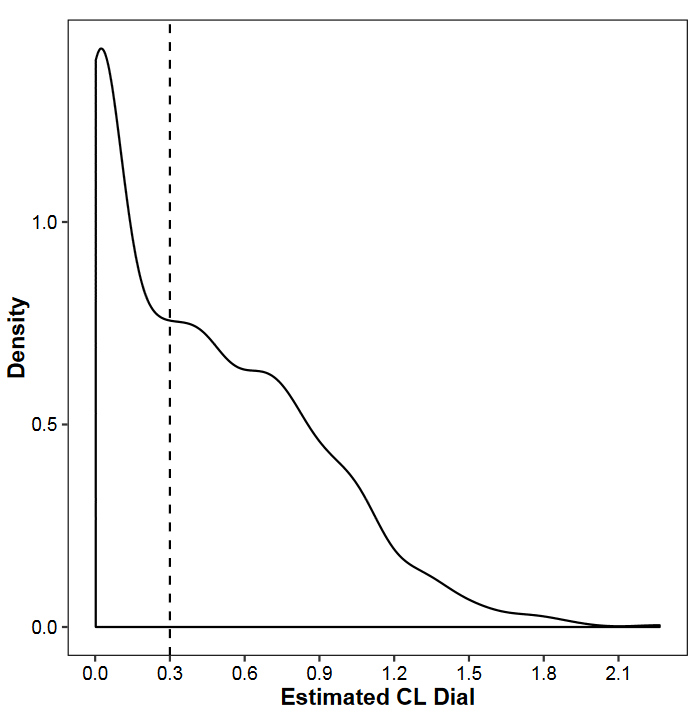


**Supplementary Figure S4:** Density of the estimated RRT clearance in clinical trial simulations using the reduced model with a body clearance of 3 L/h. Dashed line indicates the true RRT clearance of 0.3 L/h.


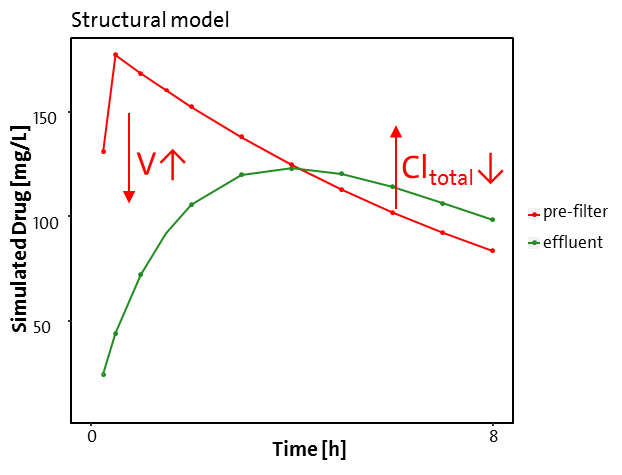


**Supplementary Figure S5** Influence of non-detected reversible adsorption to the hemodialysis membrane on the structural model when using the effluent approach.
